# A Scoping Review of Evidence-Informed Recommendations for Designing Inclusive Playgrounds

**DOI:** 10.3389/fresc.2021.664595

**Published:** 2021-05-24

**Authors:** Denver M. Y. Brown, Timothy Ross, Jennifer Leo, Ron N. Buliung, Celina H. Shirazipour, Amy E. Latimer-Cheung, Kelly P. Arbour-Nicitopoulos

**Affiliations:** ^1^Faculty of Kinesiology and Physical Education, Mental Health and Physical Activity Research Centre, University of Toronto, Toronto, ON, Canada; ^2^Department of Geography and Planning, University of Toronto, Toronto, ON, Canada; ^3^Holland Bloorview Kids Rehabilitation Hospital, Bloorview Research Institute, Toronto, ON, Canada; ^4^Rehabilitation Sciences Institute, University of Toronto, Toronto, ON, Canada; ^5^The Steadward Centre for Personal and Physical Achievement, University of Alberta, Faculty of Kinesiology, Sport, and Recreation, Edmonton, AB, Canada; ^6^Department of Geography, Geomatics and Environment, University of Toronto Mississauga, Mississauga, ON, Canada; ^7^Cancer Research Center for Health Equity, Cedar-Sinai Medical Center, Los Angeles, CA, United States; ^8^Department of Medicine, University of California, Los Angeles, Los Angeles, CA, United States; ^9^School of Kinesiology and Health Studies, Queen's University, Kingston, ON, Canada

**Keywords:** inclusive playgrounds, playground design, childhood disability, play, accessibility

## Abstract

**Background:** Playgrounds provide children with many sensory, motor, and socioemotional experiences that are critical to child development. Unfortunately, playgrounds also represent an environment where children with disabilities experience barriers to accessing play. Structures and materials that are prominently found in almost all playground designs (e.g., swings, slides, sand) can present as obstacles for many children with disabilities to engage in independent play.

**Aims:** This scoping review engaged in the empirical literature to address the research question, “What are the evidence-informed recommendations for designing inclusive playgrounds to enable participation for children with disabilities?” Consideration was given not only to the physical design of playgrounds, but also the playgrounds' surrounding built and social environments.

**Methods:** A systematic search of Medline, PsycINFO, CINAHL, EMBase, ERIC and Scopus was conducted. Only peer-reviewed literature published in English between January 1990 and January 2021, with a primary focus on inclusive playground structure design related to any type of disability were included. Data extraction included the study author(s), year of publication, country of origin, purpose, disability types considered, methods, sample characteristics and key findings. Key findings were synthesized into evidence-informed recommendations, which were later collated, using inductive content analysis, into five broader thematically congruent groups.

**Results:** Thirty-five studies were included using case study (*n* = 17); observational (*n* = 6); survey (*n* = 5); experimental (*n* = 4); and multiple study (*n* = 3) designs. Thirteen evidence-based recommendations and one promising practice were categorized into five broad playground elements: entry points; surfacing and paths; features to foster inclusive play; staffing/supervision; and design process.

**Conclusion:** These recommendations build upon previous design-based best-practices that focused exclusively on the physical design of the playground. Our recommendations have implications for how future playgrounds should be designed to maximize usability and inclusiveness and the overall playground experiences for children with disabilities.

## Introduction

It has been over 30 years since the United Nations Convention on the Rights of the Child formalized play as a fundamental human right of all children (1). The more recent United Nations Convention on the Rights of Persons with Disabilities offers further support for children with disabilities regarding equal access to play (2). Despite these declarations of human rights, the United Nations has acknowledged that the unique needs, interests, and rights of children with disabilities have continued to be overlooked, including those concerning equal access to play opportunities (3). This oversight is troubling given that play is integral to children's cognitive, physical, and social development, and to their emotional well-being (4–7).

Schoolyards and parks are an integral part of the larger experience of play within children's communities. Playgrounds—defined in this article as constructed play areas that contain traditional play equipment (e.g., swings, slides, merry-go-rounds) on the ground as well as structures built with paths to and between elevated play equipment (8)—are omnipresent within the landscape design of these spaces and constitute a significant part of the overall play experience available to children. Unfortunately, playgrounds also represent an environment where children with disabilities experience barriers to accessing play (9–16). Although playgrounds are designed to provide children with an array of opportunities to engage in different types of play, the designs are frequently informed by normative understandings of children's bodies, mobilities, and abilities that do not adequately account for the presence of childhood disability. The resultant playground designs can create inequitable access to play opportunities and may cause children with disabilities to experience exclusion (17–19).

While children with disabilities continue to experience barriers to accessing play opportunities across playgrounds, their needs are beginning to be recognized in playground design research and practice as the concept of inclusive play continues to develop (20). Inclusive play and, correspondingly, inclusive playgrounds, are intended to remove physical and social barriers to participation through designs that provide an environment where all children can play together using the same equipment (20). We conducted a scoping review of the nascent literature on inclusive playground design to contribute an updated, comprehensive analysis that can inform scholars and practitioners in designing playgrounds to enable and include children with disabilities. Our review engaged the following research question: “What are the evidence-informed recommendations for designing inclusive playgrounds to enable participation for children with disabilities?” By engaging this question, our scoping review aims to identify key playground design factors that have been found to improve play equipment usability and overall playground experiences for children with disabilities. Prior to presenting our scoping review process, results, and discussion, we briefly discuss three topics to provide necessary context: (i) playground experiences for children with disabilities; (ii) playground design standards; and (iii) past playground reviews.

### Playground Experiences for Children With Disabilities

Considering playground play is largely unstructured, it gives children opportunities to advance their imagination, self-awareness, risk perception, and identity, as well as their social and motor skills (17, 18). The diverse play opportunities that can emerge within playgrounds make these spaces a unique setting where children can choose when, where (i.e., in relation to specific equipment), and how to interact with others while playing together or sharing the play space. Although children with disabilities value playgrounds as play spaces (9, 17, 21), playground designs often do not provide them with equal and equitable access to play opportunities (9, 10, 12, 13, 15, 16). Hence, children with disabilities can face numerous barriers when trying to access playground play. Sometimes, these barriers are encountered at playground entrances (e.g., raised borders). This can make it difficult or impossible to access the playground space—let alone its play equipment—without assistance from caregivers (19, 22, 23). Further, the absence of ramps from elevated play structures can restrict some children with disabilities from accessing and moving freely on the structures (10, 13, 19). In the rare cases where elevated play structures include ramps, the structures' accessible routes can terminate at dead ends that do not provide play opportunities, access, or egress (21, 24).

In addition to issues concerning playground surfaces and elevated play structures, the play components (e.g., slides, merry-go-rounds) themselves are often inaccessible. In fact, adapted play components that enable children with physical disabilities to fully and safely engage in playground play (e.g., slides that can be accessed via ramps, wheel-on merry-go-rounds) remain largely absent from playgrounds (9, 10, 14, 15, 17). Many playgrounds also lack sensory-based play components that may promote active engagement among children with developmental disabilities, such as tactile play components that offer different textures to touch and manipulate, or musical play components that produce a variety of sounds and vibrations (25, 26). These ongoing playground design issues may explain why playgrounds have been identified as landscapes where children with disabilities can end up feeling isolated, excluded from peer interaction, or excluded from the play space entirely (17–19).

### Playground Design Standards

In North America and beyond, there are various accessibility standards that apply to playgrounds (e.g., Americans with Disabilities Act Standards for Accessible Design, Canadian Standards Association Standard for Children's Playspaces and Equipment, and Australian Standards' “AS 4685 Playground Equipment and Surfacing” and “AS 1428 Design for Access and Mobility”). These standards support the implementation of access ramps to elevated components, the provision of accessible play components that are at ground level and elevated, and the removal of barriers from playground entrances and pathways (27). Although the presence of standards represents a shift toward improving access to play opportunities for children with disabilities, the standards include limitations and do not necessarily ensure inclusive play opportunities.

One notable limitation is that playground accessibility standards are largely informed by the opinions of playground designers that have outpaced (and are thus no longer informed by) scientific evidence (28). Additionally, playground accessibility standards have often focused on addressing barriers for children with mobility impairments more so than barriers for children with sensory or developmental disabilities. As a result, playground designs often prioritize play for children with mobility impairments (25) and disregard the play of children with sensory or developmental disabilities. More empirical research, including the voices of parents and children experiencing disability, on accessible and inclusive playground design that will inform playground standards is sorely needed.

How practitioners engage and treat accessibility standards is another key concern. For example, by treating minimum accessibility standards uncritically as fixed accessibility standards (i.e., by not carefully assessing if minimum standards should be exceeded to suit a specific site and its users' needs), practitioners may produce technically accessible landscapes that meet legal requirements that are functionally inaccessible to some (29). This uncritical treatment of standards remains a concern as municipal employees who work in or in relation to parks and playgrounds have expressed having limited knowledge about inclusive design beyond addressing accessibility (e.g., adding ramps where required) and that they have no available standards for reference (10).

### Past Playground-Related Reviews

Our review builds upon two past reviews by Moore and Lynch (28) and Fernelius and Christensen (20). Moore and Lynch (28) conducted a scoping review of 14 studies that explored the accessibility and usability of playgrounds for children of all abilities. Their overarching recommendation was that the Principles of Universal Design (Center for Universal Design, 1997) should be considered when designing playgrounds to promote inclusion through equal and equitable access to play options for all children, including those with disabilities. The suggested principles would help to ensure: (i) equitable use, (ii) flexibility in use, (iii) intuitive use, (iv) provision of perceptible information, (v) tolerance for error, (vi) minimal physical effort to access, and (vii) appropriate size and space for approach and use (30).

Fernelius and Christensen's (20) review of 22 studies identified 10 specific physical design elements to improve playground play for children with disabilities. These design elements support the use of: (i) circular playground design, (ii) common and recognizable objects, (iii) loose parts, (iv) accessible surfacing and sufficient space, (v) elevated and ground level components, (vi) multi-niche settings, (vii) equipment that provides appropriate levels of challenge and risk, (viii) observation points, (ix) comfortable places, and (x) sensory stimulus.

While these two past reviews have identified ways to improve a playground's physical design in order to create play opportunities for children with disabilities, their focus has primarily been on the playground structure itself, therefore there may be additional evidence-informed recommendations to consider. For example, since playgrounds are inherently a social experience for children, and are not experienced in isolation from their surroundings, it is sensible to consider ways in which a playground's surrounding built and social environments can enhance playground experiences for children with disabilities. We engage this gap in this scoping review by considering playgrounds' physical designs, social environments, and surrounding built environments. Our intent in expanding our scope in this way is to help readers begin to move past creating play opportunities for children with disabilities through just physical design elements within the borders of the playground toward a more comprehensive approach focused on ensuring children with disabilities and their families experience inclusion during playground visits.

## Materials and Methods

Scoping reviews are a rigorous and transparent approach for synthesizing evidence when the purpose is to capture the relevant literature on a topic, regardless of the study design (31, 32). Our review follows the five recommended stages identified within existing frameworks for conducting a scoping review (31–35): (i) identifying the research question; (ii) identifying relevant studies; (iii) study selection; (iv) charting the data; and (v) collating, summarizing, and reporting the results. The following sections provide further details on stages ii–v of this review process.

### Identifying Relevant Studies

The research team developed the initial search strategy in consultation with an academic librarian. The search strategy was intentionally broad to maximize coverage of all relevant studies (35). This involved using search terms related to playgrounds: “playground^*^,” “playspace^*^,” “play space^*^,” “playscape^*^,” “play component^*^,” “play area^*^,” “play structure^*^,” “play park^*^,” and “play environment^*^.” For this review, the term “playground” refers to play areas built as part of schoolyards or parks that contain traditional play equipment (e.g., swings, slides) at ground level and structures built with paths to and between elevated play equipment (8). Disability theory and research, and hence the concepts and terms used to describe and understand disability, have evolved considerably over the past three decades. Only using playground-related search terms circumvented the potential to exclude articles that have used a variety of terms to describe different disabilities (e.g., blind vs. visually impaired) and disability design (e.g., accessible, inclusive, universal, barrier-free).

After identifying journal databases in consultation with an academic librarian, the first author conducted literature searches across Medline, PsycINFO, CINAHL, EMBase, ERIC, and Scopus databases, from January 1990 to July 2019, which was later updated in March 2020 and again in January 2021. All captured search records were exported into an online review management system that identified and removed duplicate records from the database (Covidence, Veritas Health Innovation, Melbourne, Australia). The first author completed a subsequent search of all included articles' reference lists after the full-text screening stage and the reference lists of excluded position papers and reviews to identify any additional relevant articles.

### Study Selection

Study inclusion criteria are provided in Table 1. Given that scoping reviews involve an iterative process rather than the linear process adopted by systematic reviews (31), the four-person study selection team (DB, KAN, TR, JL) regularly discussed criteria during the search process and modified them (i.e., to the criteria in Table 1) as the nature of the literature became apparent. Manufactured play structures were focused on due to the inherent differences that exist between manufactured and natural playgrounds in terms of their affordances and play opportunities (36).

**Table 1:** Scoping review study inclusion and exclusion criteria.

**Inclusion criterion**	**Exclusion criterion**
(1) Primary focus on inclusive playground structure design;	(1) Not a primary data collection study (e.g., position paper, review);
(2) Focus on disability (any type);	(2) The full text could not be obtained;
(3) Primary peer-reviewed studies of qualitative, quantitative, or mixed study design in order to consider different findings that have the potential to inform inclusive playground design practices;	(3) Playground was defined in an alternative context (e.g., an environmental playground of bacteria);
(4) Written in English;	(4) Focused on natural playground design (e.g., garden, forest);
(5) Published since 1990^*^	(5) Focused on playground injury epidemiology;
	(6) Focused strictly on playground design for safety

* *A January 1990 search start date inclusion criterion was used to acknowledge (and to capture changes in best practice recommendations since) the passing of the Americans with Disabilities Act (ADA)*.

The study selection team began screening by applying inclusion/exclusion criteria to the titles and abstracts of 100 randomly selected records (~1%). Once completed, they discussed decision discrepancies to support reliability during the screening process (37). Next, the four reviewers independently screened the titles and abstracts of the remaining 99% of records. Each record was screened by two reviewers and inconsistent decisions were resolved by a third reviewer. Upon completing the title and abstract screening stage, the first author retrieved the full-text articles for all records that met inclusion and exclusion criteria. Each full-text article was screened by two reviewers (DB, KAN) to further determine whether it should be included for review. Again, inconsistent decisions were resolved by a third reviewer (JL). Reasons for exclusion were recorded at the full-text screening stage.

### Charting the Data

The research team collectively determined which attributes of the articles to extract for summary and analysis after piloting the Microsoft Excel-based data charting form with a representative sample of the studies to be reviewed. The finalized data charting form was developed for extraction of the following study attributes: author, year of publication, country of origin, purpose, disability types considered, methods, sample and key findings. The first author independently extracted and charted the data from each article. The senior author (KAN) checked the extractions and updated the data charting form in an iterative process.

### Collating, Summarizing, and Reporting the Results

The research team summarized and reported the key findings that emerged from the charting process. The first author then synthesized the key findings into potential evidence-informed recommendations using an inductive content analysis approach (38). This approach involved applying codes to the key findings to reduce and group data into mutually exclusive concepts (recommendations). Next, the research team reviewed and revised the coding for the potential evidence-informed recommendations and further reduced and grouped the data, which were later collated into five broader thematically congruent groups (i.e., playground elements). Given the broad range of key findings identified in each study, each article could be mapped to multiple recommendations based on its contents. Next, three authors (DB, KAN, JL) independently reviewed the recommendations and playground elements prior to discussing as a team until consensus was achieved. Through team discussion, the recommendation with only one study for support was relabelled as a promising practice and identified as an area for future research.

## Results

### Selection of Studies

**Figure 1:**
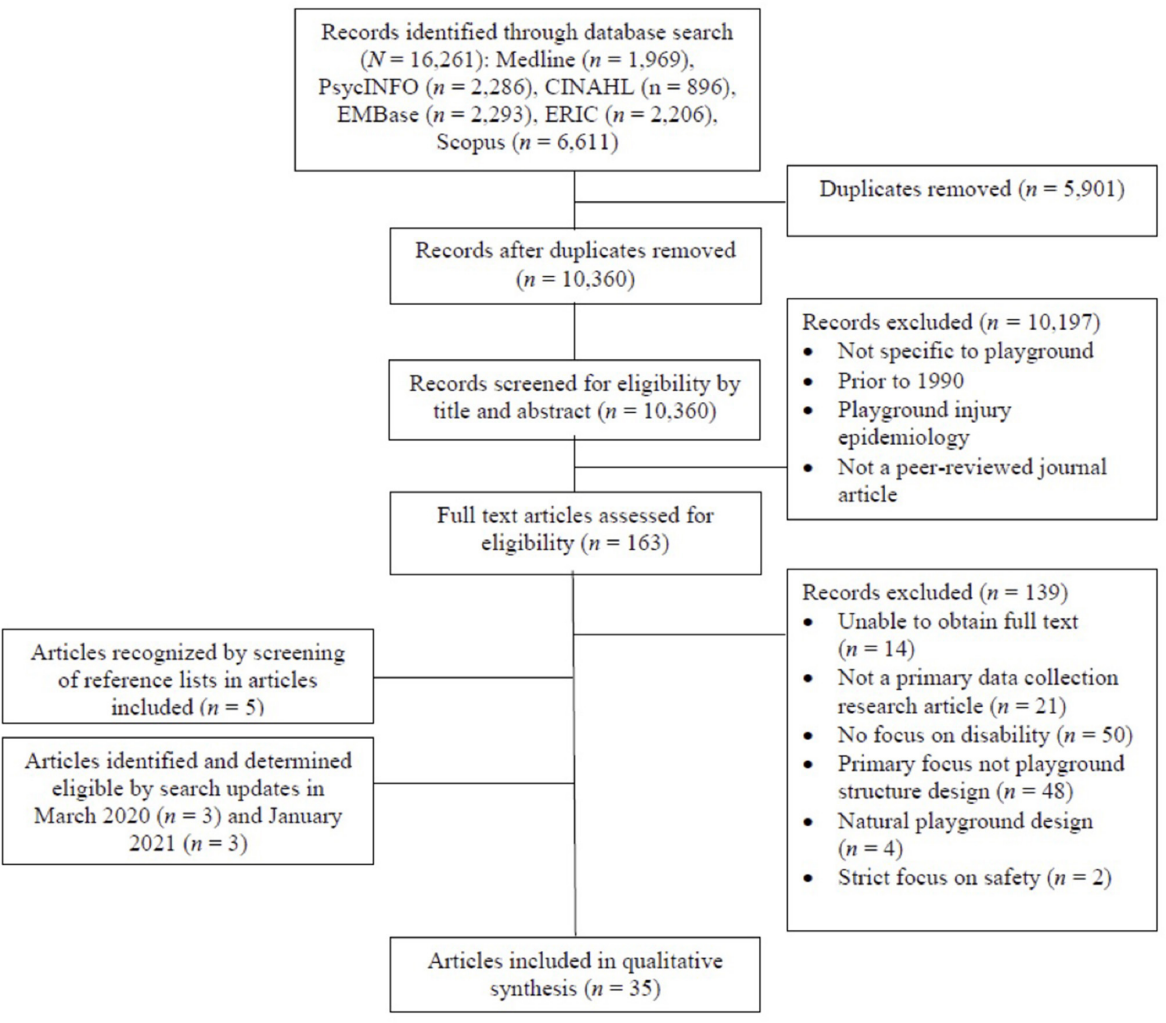
PRISMA flow chat.

The search yielded 16,261 records which was reduced to 10,360 after duplicates were removed. After screening the title and abstract of each record using the inclusion/exclusion criteria, 163 articles remained and their full texts were obtained. Full-text screening for inclusion was completed independently by two reviewers, resulting in 139 records being removed and 24 records being selected for inclusion. Reference lists of these 24 articles were screened for missed records, resulting in an additional five articles. Updated searches using the original search strategy were conducted in March 2020 and January 2021, providing an additional six articles to be included (three articles in each respective update). Overall, a total of 35 articles were selected for full review (see Figure 1 for PRISMA flow chart).

### Study Characteristics

The country of origin, purpose, disability types considered, methods, sample and key findings for each study are presented in Supplementary Table 1. Two studies were published in the 1990s (1996, *n* = 1; 1999, *n* = 1), six studies were published from 2000 to 2009 (2000, *n* = 1; 2001, *n* = 2; 2006, *n* = 2; 2007, *n* = 1), and the remaining 27 studies were published from 2010 to 2020 (2010, *n* = 3; 2011, *n* = 2; 2012, *n* = 4; 2015, *n* = 2; 2016, *n* = 2; 2017, *n* = 3; 2018, *n* = 5; 2019, *n* = 1; 2020, *n* = 5). Research was conducted in Australia (*n* = 4), Brazil (*n* = 1), Canada (*n* = 3), Hong Kong (*n* = 1), Israel (*n* = 1), the Netherlands (*n* = 1), New Zealand (*n* = 1), Poland, (*n* = 1), Sweden (*n* = 4), Switzerland (*n* = 1), Turkey (*n* = 2), United Kingdom (*n* = 3), and the USA (*n* = 12). The studies used a range of different designs including case studies (*n* = 17), observational (*n* = 6), survey (*n* = 5), experimental (*n* = 4) and multiple study designs (*n* = 3).

### Sample Characteristics

Twenty-five studies involved data collection with human participants, with sample sizes ranging from one to 667 participants. These studies included children with and without disabilities, parents/caregivers of children with and without disabilities, school staff, municipal employees, playground designers, and not-for-profit organization representatives. Studies primarily focused on children with varied disabilities (*n* = 15) and physical disabilities (*n* = 8), although one study focused on children diagnosed with autism spectrum disorder (ASD; *n* = 1) and two studies did not specify the disability focus (*n* = 2). Specific impairments represented in the 25 studies included physical (e.g., lower extremity prosthesis, cerebral palsy, mobility impairments), learning, developmental, sensory processing (e.g., ASD), visual (e.g., congenital blindness, severe visual impairment), auditory, and intellectual impairments/disorders. Among the 11 studies focused on playground audits or testing of playground surfaces, sample sizes ranged from one to 355. These studies focused on playground surfaces (*n* = 2) and audits of playgrounds at schools or parks (*n* = 6), boundless playgrounds (*n* = 1), adapted playgrounds (*n* = 1) and playgrounds at schools for children with disabilities (*n* = 1). One study involved data collection with human participants and an audit on the infrastructure of the playground which explains why 36 studies are described in this section.

### Evidence-Based Recommendations

Our analysis of each study's key findings identified 13 evidence-based recommendations for designing inclusive playgrounds for children with disabilities. One additional promising practice was identified, based on findings from one study. These recommendations and the promising practice were classified into five broad playground elements: entry points; surfacing and paths; features to foster inclusive play; staffing/supervision; and design process. A summary of the recommendations for each playground element is provided below. Table 2 provides an overview of these evidence-informed recommendations.

**Table 2:** Summary of the evidence-informed recommendations and supporting evidence for designing inclusive playgrounds for children with disabilities.

**Playground element**	**Recommendation**	**Supporting evidence**
1. Entry points		
	1.1. Entrance to the playground space is wide and free of any obstacles	(12–14, 16, 19, 25, 39, 40)
	1.2. Wide, flat and firm pathways from the entrance to the playground	(10–15, 22, 39, 41, 42)
	1.3. Enclosing the playground to prevent children from straying (*Promising Practice*)	(12)
2. Surfacing and paths		
	2.1. A flat uniform surface that consists of material that is moderately firm and stable	(11–19, 21, 23, 24, 39, 41, 43–47)
	2.2. Ramps that provide access to and between elevated play components	(10–13, 19, 21, 24–26, 39–42, 45, 48, 49)
3. Features to foster inclusive play		
	3.1. Play equipment accessible to all children	(9, 10, 12–17, 21, 22, 24–26, 39–42, 47, 48, 50–53)
	3.2. Variety of play equipment that provides appropriate challenges for children of all ages and abilities	(10, 17, 19, 21, 24, 39, 42, 46–48, 50, 53)
	3.3. Different types of sensory play components that are spread out within the play space to reduce overstimulation	(12, 21, 25, 26, 39, 41, 42, 53, 54)
	3.4. Solitary play components for escaping overstimulation	(12, 17, 39, 41, 42)
	3.5. Play components shaped in recognizable designs that allow for creative and imaginative pursuits	(17, 25, 50)
	3.6. Informational features to aid with spatial orientation, communication and guidance on proper use of equipment	(10, 12, 17, 25, 26, 39–42)
	3.7. Shaded spaces to aid with temperature regulation	(11, 21)
4. Staffing/Supervision		
	4.1. Trained staff present in the play space to support play for all children	(9, 14, 24, 40, 48, 55, 56)
5. Design process		
	5.1. User involvement (families of children with disabilities and representatives from disability organizations) in the design process	(13, 14, 21, 46, 47, 52, 55)

#### Entry Points

This playground element concerns the playground's perimeter, including any entrance, and the paths that provide access to the playground surface. Two evidence-informed recommendations were identified for entry points: (i) ensure playground entrances are wide and free of obstacles; and (ii) provide wide, flat and firm pathways leading to the playground. An additional promising practice (based on findings from one study) was identified for the entry points playground element–enclose the playground to prevent children from straying.

##### Wide, Flat and Firm Pathways Leading to The Playground

The importance of having wide, flat and firm pathways leading to a playground was highlighted in 10 of the 35 studies (10–15, 22, 39, 41, 42). Interviews with playground users and municipal playground personnel have shown children with disabilities, particularly those with mobility impairments, often cannot access the playground without assistance (13). Obstacles limiting movement into the playground include paths that have irregularities (13, 14, 22) and/or unstable ground cover, such as sand or pea gravel (12–14). To improve access to playgrounds, all paths should be wheelchair accessible (10, 41, 42) and wide enough to allow unobstructed movement into the play space (41, 42). Paths wide enough for two wheelchairs to move side-by-side have been recommended (42). While existing accessibility standards (e.g., ADA Standards for Accessible Design) mandate the availability of accessible pathways, playground audits indicate accessible pathways are uncommon (11, 15, 39), and in cases where accessible paths are present, they are often too narrow (12).

##### Playground Entrances That Are Wide and Obstacle-Free

Having a wide playground entrance that is clear of any obstacles was also found to be important [eight of 35 studies; (12–14, 16, 19, 25, 39, 40)]. Although studies have suggested that these entrances should be wide enough to accommodate those using mobility devices (25, 40), playground users and municipal playground personnel have indicated via interviews and surveys that this is often not the case (13, 14, 39). Raised playground borders (12, 19) and bollards positioned in the middle of entrances are two examples of obstacles known to make entering playgrounds more difficult for children who use mobility devices (16).

##### Enclosed Playground Space to Prevent Children From Straying

One study (12) highlighted the importance of enclosing the playground space to prevent children from straying. In a study examining the accessibility and usability of 21 parks in New Zealand, only two of the playgrounds observed had appropriate fencing (>1.2 m high) (12). The authors suggested that enclosing the playground with fencing may help keep children prone to straying, such as those with autism spectrum disorder (ASD), within the play space and away from potential hazards (e.g., open water, roads).

#### Surfacing and Paths

This playground element refers to the surface on which the play components are installed and the paths onto and between elevated play structures. Two evidence-informed recommendations emerged for surfacing and paths: (i) use a flat, uniform surface that consists of material that is moderately firm and stable; and (ii) incorporate ramps that provide access to and between elevated play components.

##### Flat, Uniform Playground Surface

Of the 35 studies, 19 (11–19, 21, 23, 24, 39, 41, 43–47) highlighted the importance of using a flat, uniform surface constructed from material that is moderately firm and stable. Children with mobility impairments have expressed that many playground surfaces impede their access to play equipment (18). Using unstable or overly soft ground cover such as pea gravel, engineered wood fiber, and sand may pose the greatest barrier to playground use for children with mobility impairments (14, 16, 17, 19, 21, 23, 24, 45–47). Poor surface material choices may limit independence, with children using mobility devices requiring assistance from a teacher to join their peers at the playground equipment (24). Playground audits in different countries have revealed most playgrounds have surfacing that fails to provide accessible routes to and between play equipment (11, 12, 15, 19, 39). Parents of children who use a lower extremity prothesis have reported that firm, flat surfaces are the easiest for their child to navigate (45). In fact, two experimental studies comparing different types of playground surfaces have demonstrated that poured-in-place rubber may be the ideal surface, as its moderately firm and stable profile promotes safety and accessibility (43, 44), thus enhancing usability and participation. Caregivers and children with disabilities have reiterated the importance of using a firm, shock-absorbent playground surface that provides an ideal blend of accessibility and safety (41).

##### Ramps That Provide Access to and Between Elevated Play Components

The second recommendation concerns the importance of having ramps that provide access to and between elevated play components [16 of 35 studies; (10–13, 19, 21, 24–26, 39–42, 45, 48, 49)]. A study involving parents of children with disabilities revealed that their children view being on the elevated play structure as more fun than ground-level activities (26). Aside from playgrounds purposely designed with accessibility in mind (25), most playgrounds lack ramp systems that provide access to elevated play structures (10–13, 19, 39). This is problematic given that stairs and ladders—equipment commonly used to provide access to elevated components—may be inaccessible and unsafe for children with mobility and visual impairments (40, 45). In addition to providing ramp access to elevated play structures (21, 41, 42), playground designers should do more to ensure there are inclusive play opportunities available on the accessible elevated play structures, as some have been found to provide no such options and, sometimes, they simply lead to dead ends (21, 24). Incorporating looped paths into accessible elevated play structures may facilitate play by enabling children to move continuously throughout the structure (48, 49).

#### Features to Foster Inclusive Play

This playground element refers to play components that account for the variety of needs, abilities, and desires of children with disabilities to facilitate quality play experiences and overall participation in playgrounds. We identified seven recommendations for features that foster inclusive play: (i) implement play equipment that is accessible to all children; (ii) ensure a variety of play equipment that provides appropriate challenges for children of all ages and abilities; (iii) provide and spread out different types of sensory play components across the play space to reduce overstimulation; (iv) offer solitary play components for escaping overstimulation; (v) implement play components shaped in recognizable designs that allow for creative and imaginative pursuits; (vi) incorporate features to aid spatial orientation, communication and guidance for using play space; and (vii) provide shaded spaces to aid body temperature regulation.

##### Play Equipment Accessible to All Children

Having play equipment that is accessible to all children was highlighted most often among features to foster inclusive play [23 of 35 studies; (9, 10, 12–17, 21, 22, 24–26, 39–42, 47, 48, 50–53)], as widely accessible equipment promotes inclusion (9, 52) and fosters interaction between children with and without disabilities (50, 53). Children with disabilities and parents of children with disabilities both voiced a desire for playgrounds that offer adapted equipment often not found at conventional playgrounds that meets their child's needs, abilities and interests (10, 47). However, studies have found a lack of specialized equipment (e.g., wheelchair accessible swings) for children with disabilities across playgrounds (12, 13, 15, 22, 24, 26, 39, 40, 52, 53). Traditional types of playground equipment (e.g., slides, swings) are generally inaccessible to some children with disabilities without caregiver assistance (14), and the work of physically transferring a child becomes increasingly difficulty as children grow older and heavier (10, 16). Shapiro (42) highlighted the importance of designing play components that can be accessed independently or with minimal transfer work, but evidence suggests that such equipment remains rare on playgrounds (51). Research also indicates that intuitive, easy-to-use playground equipment may be enabling to children with developmental disabilities (17). Multiple studies have recognized the importance of designing playground equipment and layouts such that they offer adequate space for children using mobility devices to maneuver onto and use equipment with ease (17, 25, 39, 40). Having a playground design that can accommodate the presence of adults can be helpful for having assistance readily available (48).

Some examples of the adapted equipment in the literature include raised sandboxes that accommodate children using wheelchairs (25, 39, 41), merry-go-rounds with ramped or flush surface access (15), swings with full body support (12), and static-free roller slides that can be used by children with cochlear implants without discomfort (42). Children with disabilities have identified swings as a favorite piece of play equipment (17, 21). Playground designers should be encouraged to include various swing types and sizes to support the inclusion of children with specific needs (e.g., wheel-on swings, full body support swings) and children or youth who are larger (17).

##### Variety of Play Equipment That Provides Appropriate Challenges

Twelve studies (10, 17, 19, 21, 24, 39, 42, 46–48, 50, 53) recognized the importance of having a variety of play components that provide appropriate challenges for children of all ages and abilities. Having a diverse range of play components provides children with opportunities to self-select activities that match their abilities and interests (42, 48), while also helping to promote several important aspects of healthy development [i.e., social emotional, perceptual motor, physical, intellectual, sensory; (39)]. Notably, one study found school playgrounds have limited diversity in terms of the play opportunities available to children with physical disabilities due to a lack of accessible equipment (19). Although some play components may have limited inaccessibility, children with disabilities have also voiced their enjoyment of observing other children challenge themselves when using play equipment such as climbing walls (47). For families of children with varying abilities, these findings have important implications pertaining to whether or not these families can go to playgrounds to play together. Playgrounds have also been found to lack developmentally appropriate play components for older children with disabilities (10, 21) and children without disabilities in general (10, 17, 24, 46, 53).

##### Different Types of Sensory-Based Play Components

Nine studies (12, 21, 25, 26, 39, 41, 42, 53, 54) highlighted the importance of having different sensory-based play components within playgrounds, including musical elements (12, 25, 26, 39, 41, 42, 54), tactile play components (21, 25, 26, 39, 42), and visual stimuli (39, 42). Including sensory elements is important for engaging children with sensory processing disorders and visual impairments on playgrounds (26, 53, 54). These sensory-based components should be spread throughout a playground to help prevent experiences of overstimulation among children with sensory processing disorders (42).

##### Solitary Play Components for Escaping Overstimulation

Five studies (12, 17, 39, 41, 42) called for solitary play components to offer escapes from overstimulation. These solitary play components are helpful to those children with disabilities who desire quiet, private places within a playground where they can relax away from adults (17, 41). Shapiro (42) proposed that areas of solitude may also provide children with disabilities with a sense of security. Despite the importance of areas for escaping overstimulation, play components that provide solitary spaces are rarely, if ever present at playgrounds (12, 39).

##### Play Components Shaped in Recognizable Designs

The importance of having play components shaped in recognizable designs (e.g., like a car) that foster creative and imaginative pursuits was evident in three studies (17, 25, 50). Two studies involving children with varied impairments revealed a desire for play equipment shaped in recognizable designs such as cars or houses (17, 50), although playground designers should ensure that these imaginative play components are spacious and wheelchair accessible (25).

##### Informational Features to Aid With Spatial Orientation, Communication and Guidance on Equipment Use

Nine studies (10, 12, 17, 25, 26, 39–42) highlighted the importance of informational features that aid in spatial orientation, communication, and guidance on equipment use to enhance the play experiences of children with disabilities. For instance, having a central auditory or visual cue such as a waterfall may assist with spatial orientation (42). Relief maps can provide important three-dimensional information for wheelchair users about the playground landscape (39). Despite evidence suggesting a lack of use (10, 12, 17), contrasting colors can also be used to help with spatial orientation by demarcating changes in surfacing (e.g., stairs) and potentially dangerous zones (e.g., areas around swings). An audit of playgrounds in Hong Kong revealed limited informative signage (40), although site maps and sign walls were desired to help children with disabilities understand the play components and to allow them to express their interests (41, 42). Signage should be presented in multiple formats (e.g., photos/diagrams, braille description of play component usage) to meet the diverse needs of children with disabilities (41), and to assist parents in supporting their child with navigating the playground space (17). Braille play elements can be invaluable for children with visual impairments and should be incorporated into future playgrounds (25, 26).

##### Shaded Spaces

Of the 35 studies, only two recognized the importance of providing shaded spaces on the playground for children who have difficulty regulating their body temperature (11, 21). Although shaded spaces can enhance the inclusion of children with disabilities on playgrounds, an audit of 57 playgrounds revealed only 14% of them offered some type of accessible shade (11).

#### Staffing/Supervision

This playground element refers to the presence of trained staff who can supervise and assist children on playgrounds. Seven of the 35 reviewed studies recognized the importance of having trained staff present to promote playground inclusion (9, 14, 24, 40, 48, 55, 56). The presence of trained staff was acknowledged as being important to initiating play (56), as staff can help to facilitate integrated play among children with and without disabilities (55), and provide children with disabilities with physical or instructional assistance to use equipment (9, 14, 24, 48). Interviews conducted with school staff have revealed that having trained staff present is helpful for modeling appropriate behavior and supporting children in managing their emotions (56).

#### Design Process

This playground element refers to the stages involved in determining how a playground should be designed with a focus on meeting the needs of children with disabilities. Including playground users in the design process was a key recommendation that emerged for this playground element [7 out of 35 studies; (13, 14, 21, 46, 47, 52, 55)]. Despite research indicating that municipal playground personnel and the construction industry have inadequate knowledge regarding the needs of children with disabilities (14, 52), there has been a lack of representation from individuals with lived experience (e.g., children with disabilities and their caregivers) or those who work closely with children with disabilities (e.g., disability organizations, occupational therapists) when designing playgrounds (13, 14, 52). Five studies have argued that the involvement of families of children with disabilities in the design process is critical for advancing accessibility and inclusion within playgrounds (21, 46, 47, 52, 55). Gaining insight from children with disabilities about their experiences with different play opportunities available on playgrounds can provide important feedback for future design as well as retroactive adaptations aiming to optimize inclusion (52).

## Discussion

The lack of evidence-informed recommendations in inclusive playground design is a significant practice gap that municipalities and families of children with disabilities continue to call upon for further action (52). Our scoping review addresses this call to action by synthesizing the empirical evidence on inclusive playground design recommendations that consider not only the physical design of the playground, but also the surrounding built and social environments of playgrounds for children with disabilities. Thirteen evidence-informed recommendations and one promising practice emerged from our analysis of findings from the 35 reviewed studies. These recommendations consider entry into the playground space, play components to foster inclusive play, the role of trained staff within playgrounds for facilitating social inclusion, and the involvement of families of children with disabilities, in addition to rehabilitation professionals and disability organization representatives, in the playground design process. Our recommendations have implications for how future playgrounds should be designed to maximize playground usability and inclusiveness for all children.

The design of an inclusive playground's surrounding built environment should be considered by playground designers and municipalities as they work toward providing children with disabilities with opportunities to fully participate in outdoor play (2). If the built environment surrounding an inclusive playground is excluded from design considerations, the result may be that exclusionary surroundings render the playground inaccessible to many children and caregivers alike. This may of course deter families from wanting or even being able to visit the playground in the first place. Findings from this paper and others (20, 28, 57) indicate that the environmental design of entry points and surfacing and paths warrant as much care and attention as the playground itself. Barrier-free entry points are necessary to ensure easy access to the playground, particularly for children with mobility-related impairments [e.g., (13, 25, 40)]. While deemed as a promising practice, enclosing the playground space to prevent children from straying highlights the additional consideration of the playground site's proximity to hazards such as road traffic and open water (e.g., rivers, drainage ditches). Proximity to a hazard does not necessarily make a site unsuitable for an inclusive playground, as the hazard can be mitigated via design interventions (e.g., fencing, signage) or, possibly, the removal of a hazard. With many playgrounds located within schoolyards and parks, it would be useful to have the importance of playgrounds' accessible surrounding environments acknowledged among those designing, building, and servicing areas around the playground. This may help to avoid creating barriers to the playground and, perhaps, find ways to enhance a playground's surrounding environment (e.g., via well-designed and serviced parking and pathways, building nearby washroom facilities).

Once children can fully access the playground space, there are essential play component features within the playground that could enable meaningful play for children with disabilities. Existing accessibility standards and guidelines provide technical accessibility requirements or guidelines that must be followed or, at least considered, depending on the policy context. These guidelines or standards often equip designers with “general levels of usability” (58) to help ensure that children with disabilities can access play components within the designated play space. However, as was found in several of the studies included in our review, these standards and guidelines are not often engaged critically, with little consideration given to exceeding minimum requirements to promote play opportunities in playgrounds that offer equal and equitable play opportunities to *all* children, regardless of ability (2, 3). For example, under the ADA Accessibility Guidelines (see subsection 15.6.3), which was applied in several studies included in this review [e.g., (11, 39, 49)], at least 50% of elevated play components, if provided, are required to be located on an accessible route. This may limit some children using mobility devices from accessing elevated play structures and, in turn, the learning/play opportunities and peer interactions associated with such structures.

Several studies found that children with mobility impairments required assistance from adults (e.g., caregivers, teachers) to access elevated structures and their play components so that they could join peers in play [e.g., (13, 24)]. Ramping to elevated play components, as per our surfacing and path recommendation (Recommendation 2.2 in Table 2), is one way to enhance access for those children who use mobility devices, and thus lessen the labor undertaken by parents or other caregivers to facilitate play (59). Yet even with ramping, children might require transfers from mobility aids to equipment—and so having sufficient space for transfers, and play equipment that can accommodate more than one body simultaneously, should be considered. Our evidence-informed recommendations encourage developers to critically question going beyond minimum requirements as they prepare their designs by considering the quality of playground experiences for all children as well as their families. Focusing on playground designs that intentionally foster quality play experiences (e.g., autonomy, belonging, challenge, engagement) (60, 61) and create settings that enhance dignity in play (62) may create more meaningful participation for all children, not only those with disabilities.

In addition to physical design elements, our review highlights two social design elements (staffing/supervision and user engagement in design process) that may help with addressing physical and social barriers that may challenge and exclude children with disabilities from playgrounds. While the involvement of families of children with disabilities and occupational therapists in the playground design process was acknowledged by Moore and Lynch (28), we have explicitly outlined the importance of this element as a design recommendation. Having representation from individuals who are aware of the unique needs and interests of children with disabilities (e.g., the children themselves and their parents) can help with filling playground designers' knowledge gaps about experiences of childhood disability on the playground (13, 14, 21, 46, 47, 52, 55). However, as noted by van Melik and Althuizen (52), the involvement of families of children with disabilities must be balanced with the limited time, resources, and added pressures that families of children with disabilities face. Several studies also suggested that rehabilitation professionals working with children with disabilities (e.g., occupational therapists) be considered in the playground design process (14, 52). Such professionals possess the medical knowledge about different disabilities as well as the knowledge about activities that occur within a playground and what supports the children might need. Of all our recommendations, end user involvement in the design process should be considered a vital first step toward ensuring the end result is a more inclusive play space. Such involvement should go beyond simply soliciting families' playground experiences; rather, they should be encouraged to provide critical perspectives on proposed designs and how they can be improved using child-friendly practices; in other words, ask them what they want, and what they need (e.g., drawings) (63).

Our recommendation related to staffing and supervision highlights the interaction between the built environment and social inclusion. Many physical aspects of playground design, such as swings, sand, and elevated play components, assume children with disabilities will have caregiver support to aid with transfers. Yet, there tends to be an overall lack of support available at playgrounds for children with disabilities (40). Trained staff can play a critical role in filling this void through providing children with disabilities with several types of support as well as facilitating social interactions among children with and without disabilities alike. For example, trained staff can provide physical or instructional assistance to use equipment (9, 14, 24, 48), while also helping to initiate social interactions with other children through playground play (55, 56). Our recommendation for playground staffing and supervision is focused on playground programming where trained staff are knowledgeable about strategies to foster inclusive play and are cognizant of where and how liability issues can be properly addressed. Research is needed to understand how best to train and support staff, according to the play needs and preferences of children with and without disabilities, to deliver quality supervised programming that does not exclude smaller playgrounds in neighborhoods that could be very important for inclusion with peers.

While this study represents a particularly comprehensive synthesis of the peer-reviewed literature examining playground design for children with disabilities, it is not without limitations. First, studies were only included if they were published in 1990 or later. While we recognize our search was not exhaustive in that it did not capture earlier research [e.g., (64, 65)], findings prior to 1990—the year in which the ADA became law—may not reflect how this monumental change in regulations may have impacted playground design, in the United States at least. Second, we only included published studies with empirical evidence and as a result, academic literature involving clinical suggestions [e.g., for adapted swing design (66)] and unpublished work (e.g., white papers) were excluded. As such, our findings do not represent all of the available literature that may have implications for the design of playgrounds for children with disabilities. Third, our search was restricted to studies published in English, meaning that we most likely did not capture some of the inclusive playground design literature from scholars publishing in other languages, or in other parts of the world. As a result, our recommendations are largely informed by findings from research conducted in high-income countries. Fourth, very few of the included studies incorporated disability theory [e.g., social model of disability; (50, 55)] in their analyses, which limited our ability to connect our identified recommendations to the theoretical disability perspectives that may have produced them. Fifth, our scoping review was not prospectively registered in a database. Registration can help to avoid unplanned duplication and allow for comparison of reported review methods against what was planned in the protocol. Lastly, consistent with the scoping review methodology, the quality of the included studies was not appraised. While each of the included studies went through the peer review process, we recognize that there may be bias in our findings.

Overall, 13 evidence-informed recommendations and one promising practice for designing inclusive playgrounds for children with disabilities emerged from our scoping review of 35 peer-reviewed studies. Our evidence-informed recommendations are not exclusive to the playground design itself; they also recognize the importance of ensuring easy access into playground spaces, how the presence of trained staff within playgrounds may improve accessibility and inclusion, and how involving children with disabilities and their families in playground design processes can help with producing playgrounds that account for their needs, abilities, and desires. Building playgrounds that include families with children with disabilities in the design process will help to create play spaces in the community that are welcoming and inclusive for all. We anticipate this, along with exploring the role of trained staff will help communities better support all members with disabilities, thus enabling children and parents to experience play and the associated benefits of health and well-being.

## Author Contributions

DB: methodology, investigation, data curation, formal analysis, writing–original draft, and project administration. TR: methodology, investigation, and writing–original draft. JL: methodology, investigation, validation, and writing–original draft. RB: funding acquisition and writing–original draft. CS and AL-C: funding acquisition and writing–review and editing. KA-N: conceptualization, funding acquisition, investigation, validation, writing–original draft, and supervision. All authors contributed to the article and approved the submitted version.

## Conflict of Interest

The authors declare that the research was conducted in the absence of any commercial or financial relationships that could be construed as a potential conflict of interest.
