# Supplementary material for: A Scoping Review of Evidence-Informed Recommendations for Designing Inclusive Playgrounds
Source: Front Rehabil Sci. 2021 May 24;2:664595. doi: 10.3389/fresc.2021.664595 (PMC9397725; doi:10.3389/fresc.2021.664595)
Supplement: Supplementary file 1 [file Table_1.docx]

| **Supplementary Table 1.** Summary of Study Characteristics and Key Outcomes | | | | | |
| --- | --- | --- | --- | --- | --- |
| **Study** | **Purpose** | **Disability Types Considered** | **Methods** | **Sample** | **Key Related Findings** |
| Axelson & Hurley (2018)  USA | Examine accessibility properties (i.e., firmness & stability) of common playground surface materials | Not specified | Experimental | Playground surfaces (*N* = 56) including engineered wood fibre (*n* = 36), hybrid (*n* = 2), loose fill rubber (*n* = 6), natural surface (*n* = 4), paved surface material (*n* = 2), rubber solid (*n* = 6) | -Playground surfaces that are moderately firm and stable promote safety and accessibility to the greatest degree |
| Burke (2012)  Australia | Explore the play experiences of children with and without impairments on the playground | Varied (not specified) | *Case study | Children 6–10 years old with and without varied disabilities (*N* = 72, 42% female; 48% with disabilities) | -Play components that are accessible to all children foster interaction  -Children desire play components that provide appropriate challenges for all abilities  -Children with ASD desire play components that allow for imaginative play |
| Chesney & Axelson (1996)  USA | Demonstrate the feasibility of establishing performance criteria for surface accessibility based upon ramp grade work requirements | Not specified | Experimental | Playground surfaces including rubber (*n* = 1), engineered wood fibre (*n* = 2), generic cedar chips (*n* = 1), generic bark chips (*n* = 1), sand (*n* = 1), and pea gravel (*n* = 1) | -Rubber playground surfacing requires the least amount of work to navigate |
| Jeanes & Magee (2010)  United Kingdom | Explore parents’ experiences of inclusion in their communities, their involvement in leisure opportunities as a family and their family’s experiences of using a new inclusive playground | Varied (learning, developmental, physical, sensory) | *Case study | Parents of children with varied disabilities (*N* = 32 from 18 families) | -Adapted equipment fosters inclusion  -Having trained staff present on playgrounds promoted inclusion through provision of assistance to use equipment |
| Jeanes & Magee (2012)  United Kingdom | Explore parents’ and children’s experiences of using a new inclusive playground and whether it was considered inclusive | Varied (learning, developmental, physical, sensory) | Case study | Children with varied disabilities (*N* = 18; 4-12 years old at beginning of study).  Parents of children with varied disabilities (*N* = 32 from 18 families) | -Including families of children with disabilities in the design process helped to address accessibility and inclusion issues commonly found at previous playgrounds through incorporation of adapted play components that could be used by children with disabilities as well as those without  -Having trained staff present on playgrounds promoted inclusion through provision of assistance to use equipment and facilitating integrated play opportunities among children with and without disabilities opportunities |
| Kerfield et al. (2018)  USA | Explore participation in active play of children who use lower extremity prostheses | Physical | Survey | Parents of children who use lower extremity prostheses (*N* = 26) | -Firm, flat surfaces (e.g., grass, rubber) are the easiest to navigate  -Loose, uneven surfaces (e.g., sand) pose balance problems  -Play components requiring secure placement of prosthetic foot such as ladders pose increased challenges |
| Kern & Wolery (2001)  USA | Evaluate the effects of adding musical stations and a path between them to a playground | Varied (visual, developmental) | Single-subject experimental | One 3-year-old boy with congenital blindness and compromised developmental abilities | -Adding musical stations and a connecting path increased play and decreased stereotypic responses, but did not increase social interactions or movement within the playground |
| Kodjebacheva et al. (2015)  USA | Assess the design of the boundless playgrounds in terms of (i) safety and accessibility, and (ii) availability of sensory elements | Varied (physical, sensory, auditory, visual) | Observational | Boundless playgrounds (*N* = 10) | *Safety/Accessibility*  -Most playgrounds had wheelchair-accessible entrances  -Most playgrounds had sufficient turn-around space for a wheelchair as well as tables that were wheelchair-accessible  -All playgrounds had wheelchair-accessible ramps to reach elevated play components  -4/10 playgrounds had sandboxes at multiple heights to accommodate wheelchairs  *Availability of Sensory Elements*  -Playgrounds had wheelchair-accessible imaginative play areas  -6/10 playgrounds had sandboxes although 3/6 lacked a sufficient amount of sand  -9/10 playgrounds had musical and noise-making elements  -1/10 playgrounds had a Braille play component |
| Lynch et al. (2020)  United Kingdom | Understand play participation in five local playgrounds by exploring the perspectives of play providers and families with diverse abilities | Varied (physical, developmental, sensory, visual) | *Case study | Play providers (n = 4), children with and without varied disabilities (n = 12), and adults with and without varied disabilities (n = 10) | -Playgrounds lacked developmentally appropriate play components for older children  -Absence of ramps limited access to and use of play components for children with physical disabilities  -Absence of elements (e.g., brighter colours) to guide children with visual impairments away from dangerous zones  -Adapted equipment is desired for children with varied impairments  -Accessibility addressed primarily through provision of wheelchair accessible footpaths |
| Menear et al. (2006)  USA | Design a multipurpose fitness playground for children with ASD | Sensory | Case study | Playground designers; School personnel. Sample sizes not provided. | -Building a large playground allows for inclusion of a variety of play components  -Having trained staff on-site can help with providing instructions regarding play  -Provide opportunities to self-select activities to match abilities and interests  -Spacious open playground design gives staff space to stand and provide assistance  -Including a variety of play elements at different heights allows children to play according to their abilities and desired challenge  -Connecting pathways on raised equipment helps children progress continuously through the playground |
| Olgan & Kahriman-Ozturk (2011)  Turkey | Determine the status of playground areas for young children | Varied (visual, auditory, physical) | Observational | Playground areas at public (*n* = 17) and private (*n* = 17) schools | -Playgrounds were not designed to facilitate easy transfer and access for children who have varied disabilities |
| Olsen & Dieser (2012)  USA | Determine compliance of playground structures with the ADA Standards for accessible design guidelines | Not specified | Case study | Parks (*N* = 57) with playground structures | *Accessible routes*  -Only 5% had an accessible route that connected the playground equipment to the park entrance or parking area  *Accessible surfaces*  -All playgrounds had either woodchips or sand  *Play components*  -Only 14% had some type of accessible shade  *Ground level play components*  -Only 32% were compliant with the ADA recommendation of the ratio between ground-level play components and elevated play components  *Elevated play components*  -95% had either a ramp or transfer system  -Only 44% met the correct ADA requirement for ramps and transfer systems |
| Pawlikowska-Piechotka (2011)  Poland | Determine how to create a playground environment that meets every child’s needs | Varied (physical, visual, auditory, sensory) | *Case study | Pre-school children ages 3-6 years and their caregivers (*N* = 90) | *Access*  -Paths should be wide enough with firm surface to allow easy movement of wheelchairs to play equipment  -Wood surfaces and unitary materials are preferred; pea gravel and sand have poor accessibility qualities  *Surface*  -Firm, shock-absorbing and slip-proof  -Synthetic surfacing preferred, asphalt and concreate forbidden  *Playground*  -Include site maps with categories of play components presented in Braille or verbally via audio recording  -Include raised sandboxes that enable children with disabilities to participate  -Include sound components to improve experience for children with disabilities  - Include ramps to allow access to elevated structures for children with mobility impairments  -Include solitary play spaces that allow children with disabilities to relax and avoid overstimulation |
| Perry et al. (2018)  New Zealand | Evaluate the accessibility and usability of parks and playgrounds | Not specified | Observational | Parks and playgrounds (*N* = 21) | *Accessible Routes*  -Only 7 ground level play components in the 21 parks had an accessible route  -Only 8 playgrounds had accessible routes to elevated play components and of these, only 1 met the recommended dimensions (width of 0.61 m, height of platform between 0.45 m and 0.28 m, height of steps at least 0.2 m)  *Facilities and Amenities*  -Only 2 parks had fencing around the main play area that was greater than 1.2 m high  -No playground had high contrast colours to demarcate different heights and fall zones  -Only 2 playgrounds had auditory stimulation (e.g., chimes, drums)  -All parks had swings, however only 6 had a full-body support swing  -No park provided a solitary play space that allowed children and parents to avoid stimulation  -17/21 parks had at least one high curb, narrow path and/or irregular path surface that made using a mobility device more challenging |
| Pratt et al. (2016)  USA | Compare the effect of different playground environments (ADA compliant vs. non-compliant) on physical activity behaviour | Physical | Alternating treatment single-subject experimental | Children with cerebral palsy ages  7-8 years (*N* = 5; 80% girls) | -ADA-compliant playgrounds facilitated greater levels of physical activity and variety of play behaviour compared to non-ADA compliant playgrounds |
| Prellwitz & Tamm (1999)  Sweden | Explore the playground creators’ and users’ attitudes about accessibility problems in playgrounds | Physical | *Case study | Municipal playground personnel (*n* = 5)  Users of playgrounds including three children ages 7-11 years with mobility impairments, one parent, one personal assistant, and one school assistant | *Playground Creators*:  -Possess insufficient knowledge/awareness of the needs of children with mobility restrictions  -Construction industry does not possess adequate knowledge about adaptations for people with disabilities  -Lack of child representation among decision makers when designing playgrounds  *Playground Users*:  -Playgrounds are highly inaccessible due to narrow fence openings, soft surfacing (e.g., sand) and uneven ground (e.g., ditches)  -Very few play components had an accessible route  -Traditional types of play equipment (slide, roundabout and swings) are not accessible without assistance from parent/caregiver |
| Prellwitz et al. (2001)  Sweden | Investigate the accessibility of playgrounds for children with mobility impairments | Physical | Survey | Municipality playground personnel (n = 54) | Playground personnel’s perceptions regarding:  *Playground adaptations*  -Fully adapted (0.8%)  -Partially adapted (2%)  -Not adapted in any way (97.2%)  *Access to playground through entrance*  -Possible in all playgrounds (24%)  -Possible in some or most playgrounds (49%)  -Not possible at any playground (10%)  *Children with disabilities can move to play equipment independently*  -All playgrounds (2%)  -One or a few playgrounds (54%)  -No playgrounds (17%)  *Possibility for a child using a wheelchair to use play equipment*  -All playgrounds (0%)  -One or more playgrounds (46%)  -No playground (15%)  *Consultations with community when making changes to playground*  -Schools or daycares (61-71%)  -Children (37%)  -Parents (15-20%)  -Disability organizations (7-12%)  *Factors that limit playground accessibility*  -Loose ground cover (e.g., sand, pea gravel)  -Playground entrances that are too narrow or have a gate that is difficult to open  -Play equipment located at the center of play space areas with no accessible path  -Raised playground borders  -Enclosures with narrow openings  -Uneven ground  -Steep gradients  -Absence of ramps leading to elevated play equipment  -Absence of adapted playground equipment for children with mobility impairments |
| Prellwitz & Skär (2007)  Sweden | Understand how children with different abilities use playgrounds to engage in creative play and interact socially with their peers | Varied (physical, visual, developmental) | *Case study | Children ages 7-12 years with and without varied disabilities (*N* = 20, 45% girls) | -Swings identified as the most important piece of play equipment  -Children with disabilities desire private places without adults (e.g., benches, houses)  -Adequate level of challenge identified as necessary for both children with and without disabilities  -Desire for play equipment shaped in recognizable designs (e.g., like a car) for imaginative play  *Design factors that limit accessibility for children with disabilities*  -Sand was the biggest obstacle for children with restricted mobility  -Playground equipment was too small to maneuver around for those using mobility devices (e.g., entering/exiting playhouse)  -Playground equipment often constructed using grey wood which made it difficult for children with visual impairments to see stairs and barriers  -Playground equipment was complicated to understand for children with developmental disabilities (e.g., where to start and how to use)  -Swings and other equipment that were for sitting on are often too small for children with developmental disabilities |
| Ripat & Becker (2012)  Canada | Understand experiences of playground use among children with disabilities and their caregivers | Varied (physical, sensory) | *Case study | Children with varied disabilities (*n* = 4); caregivers of children with varied disabilities (*n* = 12); other caregivers who had a disability (*n* = 2); educational assistant (*n* =1); and sibling of a child with a disability (*n* =1) | -Play components that involve movement (e.g., swinging), different sensations (e.g., textured equipment) and height are valued  -Providing a variety of age-appropriate play opportunities that offer different levels of challenge is valued  -Ground surfaces such as sand, gravel, grass and boards/railway ties limit access  -Accessible surfacing, ramps and pathways promote access  -Absence of play opportunities at the top of ramps on some play structures  -Access to shade is important for children who have difficulty with temperature regulation  -User involvement in playground design is critical for promoting inclusivity |
| Rocha et al. (2018)  Brazil | Evaluate the accessibility of a school playground and analyze the participation of children with cerebral palsy on it | Physical | Observational | Children with cerebral palsy ages  4-6 years (*N* = 3) | *Access to playground equipment*  -All 4 playgrounds did not have suitable footpaths for accessing playground equipment  -All 4 playgrounds did not have a flat surface  -3/4 playground surfaces consisted of grass which met Brazilian design standards  *Characteristics of playground equipment*  -0/4 playgrounds had swings or merry-go-rounds that were accessible for children in wheelchairs  - Merry-go-round did not have a ramp for wheelchair access  -Swings did not have a backrest, footrest or seatbelt |
| Rouse et al.  (2020)  USA | Evaluate playgrounds to determine if i) they met ADA accessibility guidelines, and ii) the playground equipment had been modified so that children with and without special needs could play together | Not specified | Observational | 68 public school elementary playgrounds built after 2005 | *Layout accessibility criteria*  -No playgrounds met 100% of criteria in any of the 10 specific areas (71% met <50% of criteria):  -43% met site location  -32% met traffic patterns  -19% met play components  -15% met parking and curbs  -6% met soft contained play structures  -4% met walkways  -4% met practical aesthetics  -3% met clearance  -1% met surface treatments  -1% met accessible routes  *Playground equipment accessibility criteria*  - 5% met criteria in any of the 11 specific areas (91% met <50% of criteria):  -61% met reach ranges  -35% met clear floor/ground space  -25% met transfer steps  -24% met entry points/seats  -18% met transfer supports  -16% met transfer system  -11% met maneuvering space  -10% met elevated ramp run  -6% met landings - level surface  -6% met handrails  -4% met transfer platforms  *Adapted playground equipment targeting healthy development*  - 68% of playgrounds did not have modified equipment to target any of the developmental areas (i.e., social emotional, perceptual motor, physical, intellectual, sensory)  -23% of the playgrounds had modified equipment in one developmental area |
| Shapiro (2006)  Israel | Describe the planning and implementation of an adapted playground | Varied (physical, visual, auditory, sensory, developmental) | *Case study | Insufficient information | *Playground design*  -Differentiating playground into coloured segments with pathways and connections from one piece of equipment to another makes spatial orientation easier for children with disabilities  -Different size playground spaces provide a sense of security and belonging (e.g., smaller areas for solitary pursuits vs. large open areas for active play)  *Sensory experiences built into the design*  -Music areas provide children with opportunity to experiment with a variety of sound-making equipment and experience vibrations  -Spread out musical equipment over different areas to reduce over stimulation  -Select musical instruments that produce low tones rather than high pitched sounds  -Carefully consider colour of elements to ensure visual stimuli is not overstimulating  -Provide a variety of textured surfaces and materials to provide greater perceptual information  -Including a waterfall in the middle of the playground acted as an auditory cue for visually impaired children to find their way  -Moving water provides visual stimulation for children with sensory processing disorders  *Accessibility*  -Ensure all paths are wheelchair accessible, wide enough for two wheelchairs to move side-by-side, and have visibly different curbs to help children see and feel the edge  -Including a communication sign wall can assist non-verbal children with expressing their needs by pointing to different signs (e.g., “I want to use the swings.”)  -Ensure benches have no side rails in order to allow wheelchair sitting as an extension to the bench  -Include ramps to allow access to elevated play components  -Slide should have a 30cm by 40cm deep seat to accommodate wheelchair transfers  -Include a roller slide that produces no static electricity to ensure children with cochlear implants do not experience discomfort  -Park equipment should allow children with different abilities to discover and engage in activities that are most appropriate to their abilities |
| Sterman et al. (2019)  Australia | Understand outdoor play decision-making for children with disabilities from the perspectives and interactions of local government and families of primary school-aged children with disabilities | Varied (physical, developmental) | Case study | Mothers of children with varied disabilities ages 5-12 years (n = 5), local government employees (n = 4), not-for-profit organization representatives (n = 2) | -Ground cover such as sand limits accessibility for children using wheelchairs  -Involving local community members in the design process of a playground helps ensure the end result reflected needs of the community  -Families prefer playgrounds that are more challenging and meet the needs children with and without disabilities |
| Sterman et al. (2020)  Australia | Learn from participants about the utility of an intervention for promoting choice and control among children with disability on the school playground | Varied (sensory, intellectual) | *Case study | School staff including special education teachers (n = 12), teaching assistants (n = 3), occupational therapists (n = 2), school coordinators (n = 2), speech language pathologist (n = 1), principals (n = 2), and general education teachers (n = 4) | -Trained playground personnel were helpful for modelling how to play in an appropriate manner, supporting children struggling to manage their emotions and initiating play opportunities |
| Siu et al. (2017)  Hong Kong | Identify issues about the inclusiveness and accessibility of playgrounds in densely populated areas | Not specified | Case study | Playgrounds (*N* = 105) | -Inclusiveness of playgrounds and play spaces is not well addressed in Hong Kong as current design of playgrounds does not cater for the needs of children with disabilities  -Playgrounds had a lack of signage, guidance and assistance available  -Play components were not inclusive as stairs within playground did not allow children with physical and visual disabilities to access the slides  -Playgrounds should have an entrance wide enough to allow children on wheelchairs to enter the playground, wider passageways than typically observed in playgrounds, adequate room for children using wheelchairs to move around, and adapted equipment designed for children using wheelchairs |
| Stafford (2017)  Australia | Illustrate considerations in accessing playgrounds for children with mobility impairments and their families | Physical | *Case study | Children ages 9-12 years with diverse mobility impairments and their families (*N* = 10) | -Positioning a bollard in the middle of a path at the entry to a park is an issue for those using mobility devices  -Grass and bark surfaces are difficult for people using wheelchairs and crutches to maneuver on  -Playground components become more difficult to use as children get older and heavier because parents are no longer able to perform assisted transfers to overcome environmental barriers |
| Stanton-Chapman & Schmidt (2016)  USA | Examine perceptions and beliefs toward playgrounds in their school or community | Varied (physical, sensory) | Survey | Special education professionals (*N* = 303) | -99% believed their playground was not appropriate for their students  -93% worried about their students’ safety  -66% believed their students were not interested in the playground equipment  -53% believed their playground does not offer activities their student(s) likes  *Design factors that limit inclusion of children with disabilities*  -Playgrounds lack accessible equipment  -Surfacing (e.g., wood chips) reduces access and use of playground  -Lack of equipment that provides appropriate challenges for all children  -Some accessible components do not provide access to play opportunities (e.g., ramps to nothing)  -Children using wheelchairs often require assistance (i.e., transfer) from teacher to provide access to playground structure in order to join their peers |
| Stanton-Chapman & Schmidt (2017)  USA | Obtain input as to whether current playground equipment meets children’s needs | Varied (physical, sensory) | Survey | Caregivers of children with varied disabilities ages 2–5 years (*N* = 149) | -95% were not satisfied with current playgrounds  -65% believed the playground was not appropriate for their child  -55% reported their child was not interested in the playground  -Lack of accessible equipment for children with physical disabilities  -Ground-level activities are viewed as not being as fun as being on the equipment  *Design recommendations*  -Meet the needs of young children with sensorimotor concerns by incorporating musical and other noise making components, sand boxes and Braille play elements |
| Stanton-Chapman & Schmidt (2018)  USA | Examine what caregivers of children with disabilities desire regarding inclusive playgrounds for their children, the constraints that affect their recreational opportunities for the family as a whole, and how they would design a playground that would support families in overcoming social participation constraints | Varied (physical, sensory, developmental, intellectual, learning) | Survey | Caregivers of children with varied disabilities (*N* = 491) | -65% reported playgrounds were not appropriate for their child with a disability as no adaptations were made  -55% reported playgrounds do not offer activities their child enjoys  -35% reported concern for their child’s safety when using the playground  *Design recommendations*  -Include playground equipment that meets the needs of children with sensorimotor concerns  -Include a variety of play equipment that meets the needs of all family members (i.e., those with and without disabilities)  -Provide inclusive equipment that fosters interaction between children with and without disabilities |
| Stephens et al. (2015)  Canada | Determine i) how children with mobility impairments perceived and used their homes, schools and neighborhoods, ii) the types of barriers they experienced, and iii) their suggestions to improve the accessibility and inclusivity of these environments | Physical | Case study & cross-sectional survey | Case study: Children with mobility impairments (*N* = 13)  Survey: Children with mobility impairments (*N* = 406) | -50% reported uneven pathways that limit access to the playground  -50% reported inaccessible equipment  -Design made playground space too crowded |
| Talay et al. (2010)  Turkey | Examine physical and social barriers families of children with disabilities face using playground and to evaluate playgrounds in terms of the physical barriers causing restricted use by children with disabilities | Not specified | Observational & cross-sectional survey | Survey: Parents of children with disabilities (*N =* 667)  Observational: Playgrounds (*N* = 355) | -41.4% of ground surfaces were appropriate  -0.5% had ground surfaces consisting of sand  -46% do not meet the needs of the children with disabilities  *Reasons why respondents do not bring their children to parks/playgrounds*  -Parents viewed the playgrounds to be unsafe and inaccessible |
| Tamm & Skär (2000)  Sweden | Gain knowledge and understanding of how children with mobility impairments play in different play situations | Physical | *Case study | Children with mobility impairments ages 6-12 years (*N* = 10) | -Playgrounds not built to allow children using wheelchairs to access the playground equipment  -Certain games and play environments require assistance from adults (e.g., navigating inaccessible ground cover such as sand) |
| Van Melik & Althuizen  (2000)  The Netherlands | Examine the extent to which Dutch municipalities stimulate inclusive play in their (semi) public play areas | Not specified | Cross-sectional survey & case study | Survey: Municipal play providers (*N* = 151)  Case study: Municipal play providers (n = 7) families with disabled children (n = 2), neighbourhood association representative (n = 1), playground association representative (n = 1), play professionals (n = 3). | -64% of municipalities have playgrounds with adapted equipment to promote inclusion  -Inclusive equipment that can be used by all children reduces feelings of isolation experienced by children with disabilities when using specialized equipment (e.g., wheelchair swing)  -Municipal play providers lack knowledge of the needs of children with disabilities  -Overall lack of including children with disabilities (11%) or parents of children with disabilities (7%) in playground design  -Having children with disabilities test playgrounds can provide important feedback regarding inclusive design |
| Wenger et al.  (2020)  Switzerland | Explore the play experiences of children with and without disabilities on inclusive playgrounds | Varied (physical, visual, neurodevelopmental) | *Case study | Children with (*n* = 14) and without (*n* = 18) disabilities ages 7-12 years | -Inclusive playgrounds provide play opportunities different than those available on conventional playgrounds  -Children with disabilities have different needs such as additional handrails, extended entries and exits on slides  -Children with disabilities enjoy the physical act of climbing on play equipment or watching other children climb  -Overly soft fall protection floor hinders wheelchair mobility within playground boundaries  -Tables and seating areas should be designed to accommodate different sized wheelchairs  -Children with disabilities have unique perspectives that should be considered when designing inclusive playgrounds |
| Yantzi et al. (2010)  Canada | Examine the suitability of public school playgrounds for children with physical disabilities | Physical | Observational | Playgrounds at schools for children with physical disabilities (*N* = 5) | -One playground met all requirements and received a grade of 100%  -One playground met 2/3 requirements  -One playground met 1/3 requirements  -Two playgrounds met 0/3 requirements  *Reasons* *for not meeting requirements*: -Inaccessible elevated components limited play opportunities in four playgrounds  -Lack of ramps or transfer systems to access elevated components  -Imbalance between the quantity of elevated and ground-level components  -Quantity of accessible elevated components was the most frequently unmet requirement  -Limited diversity of activities provided by accessible components  -Four playgrounds had inappropriate surface materials (i.e., woodchips or sand) and borders (i.e., prominent wooden border around perimeter)  -One playground had an appropriate surface material (i.e., poured rubber) |
| *Note*. * Authors have inferred the study approach due to insufficient methodological information in the article. ASD = autism spectrum disorder; ADA = Americans with Disabilities Act. | | | | | |
